# FindMyApps compared with usual tablet use to promote social health of community-dwelling people with mild dementia and their informal caregivers: a randomised controlled trial

**DOI:** 10.1016/j.eclinm.2023.102169

**Published:** 2023-08-30

**Authors:** David P. Neal, Teake P. Ettema, Marissa D. Zwan, Karin Dijkstra, Evelyn Finnema, Maud Graff, Majon Muller, Rose-Marie Dröes

**Affiliations:** a Department of Psychiatry, Amsterdam UMC, Location Vrije Universiteit Amsterdam, De Boelelaan 1117, 1081 HV, Amsterdam, the Netherlands; b Amsterdam Public Health Research Institute, Mental Health Program, Amsterdam, the Netherlands; c Alzheimer Center Amsterdam, Neurology, Vrije Universiteit Amsterdam, Amsterdam UMC Location VUmc, De Boelelaan 1118, 1081 HZ, Amsterdam, the Netherlands; d Amsterdam Neuroscience, Neurodegeneration, Amsterdam, the Netherlands; e Saxion University of Applied Sciences, School of Health, Research Group Smart Health, Handelskade 75, 7417 DH, Deventer, the Netherlands; f Research Group Living, Wellbeing and Care for Older People, NHL Stenden University of Applied Sciences, Rengerslaan 8-10, P.O. Box 1080, 8900 CB, Leeuwarden, the Netherlands; g Department of Health Science, Section of Nursing Research & Education, University Medical Center Groningen, University of Groningen, Groningen, the Netherlands; h Radboud University Medical Center, Radboudumc Research Institute, Scientific Center for Quality of Healthcare (IQ Healthcare), 6525 GA, Nijmegen, the Netherlands; i Radboud Alzheimer Center, Radboud University Medical Center, 6525 GA, Nijmegen, the Netherlands; j Department of Internal Medicine, Geriatrics Section, Amsterdam UMC, Location Vrije Universiteit Amsterdam, De Boelelaan 1117, 1081 HV, Amsterdam, Netherlands; k Amsterdam Cardiovascular Sciences, Amsterdam, the Netherlands

**Keywords:** Dementia, Quality of life, Social health, eHealth, Randomized controlled trial

## Abstract

**Background:**

FindMyApps is a tablet-based eHealth intervention designed to help people learn to use a tablet and find easy-to-use apps. This study evaluated the effectiveness of FindMyApps for supporting social health of people living with dementia, and sense of competence of their informal caregivers.

**Methods:**

A single-centre, two-arm, non-blinded randomised controlled trial was conducted (Netherlands Trial Register NL8157). From 1st January 2020 to 31st July 2022, community-dwelling people in the Netherlands with a pre-established diagnosis of mild cognitive impairment (MCI) or dementia (Brief Cognitive Rating Scale 17–32), an informal caregiver and internet connection were allocated by block randomisation to receive FindMyApps or digital care-as-usual. Primary outcomes (measured at baseline and after three months) for people with dementia/MCI were self-management (Adult Social Care Outcomes Toolkit total score) and social participation (Maastricht Social Participation Profile frequency and diversity scores), and for caregivers, sense of competence (Short Sense of Competence Questionnaire total score). Between-group differences were tested by MANCOVA or ANCOVA (alpha = 0.05).

**Findings:**

150 dyads were randomised (FindMyApps n = 76, care-as-usual n = 74). Follow-up data were available for 128 dyads (FindMyApps n = 64, care-as-usual n = 64), who were included in the analysis in the trial arm to which they were assigned. No harms of the intervention were identified. There were no statistically significant differences in outcomes for people with dementia/MCI at group level. Diagnosis and experiencing apathy appeared to be relevant effect modifiers of secondary outcomes (neuropsychiatric symptoms, positive affect, sense of belonging, and pleasurable activities). Caregivers who received FindMyApps had higher sense of competence at three months (F [1,123] = 7.01, p = 0.0092, η^2^ = 0.054).

**Interpretation:**

Overall we found no evidence that the FindMyApps intervention better supported social participation or self-management of people with MCI/dementia than digital care-as-usual. FindMyApps does seem to better support informal caregivers’ sense of competence. For people with a diagnosis of mild dementia and older people, better tailored interventions, implementation and outcome measures may be needed.

**Funding:**

Marie Skłodowska Curie Actions Innovative Training Network H2020 MSCA ITN, grant agreement number 813196.

**Research in context**

**Evidence before this study**

Systematic reviews found that few eHealth interventions purporting to support social health (self-management and social participation) of people with dementia had been evaluated in RCTs and most studies were hamstrung by insufficient statistical power, lack of active controls and poor reporting. From these studies, very low quality evidence suggested that people with MCI may benefit more from eHealth interventions than those with dementia.

**Added value of this study**

This study overcame limitations of previous studies by comparing the FindMyApps intervention to an active digital care-as-usual control in an adequately powered study. On the one hand, the results demonstrate the potentially limited value of dementia-specific interventions compared to generic digital interventions. On the other hand, the significant group-level effect of the intervention on caregiver outcomes, despite a diverse sample of participants, provides some of the strongest evidence to date to support digital interventions for caregivers of people with dementia. The study provides stronger evidence that a diagnosis of MCI and younger age may predict better outcomes with eHealth interventions.

**Implications of all the available evidence**

Pending results of an economic evaluation, these results may support implementation of tablet-based eHealth interventions, including FindMyApps, in routine care, for supporting social participation of people with dementia/MCI, and sense of competence of informal caregivers. Implementation of tablets in general may be more beneficial for people with a diagnosis of MCI and younger people with mild dementia/MCI. For people with a diagnosis of mild dementia and older people with mild dementia/MCI, better tailored interventions, implementation processes and outcome measures are needed.

## Introduction

Dementia, or major neurocognitive disorder, is a major cause of morbidity and mortality for those living with the condition, and of stress and poor health outcomes for their caregivers.^1^ More than 55 million people worldwide live with dementia, which may more than double by 2050.^1^ Pathologies underlying dementia also cause mild cognitive impairment (MCI), which progresses to dementia within five years in 4–40% of cases.^2^ There is no cure for dementia and cost-effective, scalable interventions to support quality of life of people with dementia/MCI and their caregivers are urgently needed.

Quality of life in dementia depends on personal, disease-related, social and environmental factors.^3^ Social health is an essential contributor to good quality of life, and consists of three domains: capacity to fulfil one's potential and meet obligations; ability to self-manage one's own life; and ability to participate in social and other meaningful activities.^3^^,^^4^ In small-scale studies, eHealth interventions using tablet computers, wearable devices, virtual reality, social robots and software applications have been implemented with some success to support social participation or self-management.^5^^,^^6^ Very few randomised controlled trials (RCTs) with eHealth interventions have been published and most lacked active control conditions, were statistically underpowered or failed to follow best-practices in reporting.^6^ Several studies have reported difficulties with implementation of technologies in dementia care but it remains unclear which individuals may benefit most from digital interventions.^7^

FindMyApps is a person-centred, dyadic eHealth intervention which could support self-management and social participation in mild dementia/MCI, and sense of competence of their informal caregivers.^8^ FindMyApps aims to help people (learn to) use a tablet and to find apps, which are user-friendly for people with mild dementia/MCI, which may facilitate social contact (e.g. through video-calls, instant messaging or multiplayer games) or self-management (e.g. medication reminder apps and diaries), and which meet an individual's needs and interests. The intervention aims to support capability, opportunity and motivation to adopt the use of such tablet apps, which could support self-management and participation in social and meaningful activities. It is hypothesised that by better supporting self-management and social participation of the person with dementia/MCI, their informal caregiver will experience a greater sense of competence, meaning they feel more able to care effectively for their counterpart. Following the Medical Research Council framework for the evaluation of complex interventions,^9^ pilot studies demonstrated that FindMyApps is feasible to implement, can be positively experienced by people with dementia/MCI and their caregivers, and may improve the quality, if not quantity of home tablet use.^10^^,^^11^

The primary goal of this study was to evaluate the benefits of the FindMyApps intervention to people with mild dementia/MCI and their informal caregivers, compared to a digital care-as-usual control intervention. We investigated the effect of FindMyApps on people with dementia/MCI's self-management and social participation, and informal caregivers' sense of competence. Effects of the interventions on secondary outcomes (experienced autonomy, engagement in pleasurable activities, neuropsychiatric symptoms, and quality of life of the person with dementia/MCI, and experience of care and attitudes to dementia of the caregiver) were also evaluated. Finally, we aimed to identify effect modifiers and participant characteristics associated with better post-intervention outcomes.

## Methods

The study protocol has previously been published and is summarised here.^12^ CONSORT guidelines were followed in conducting and reporting on the study.

### Trial design

We performed a two-arm, randomised controlled non-blinded superiority trial, with two measurements, at baseline (T0) and three months post-intervention (T3). Participant consent, screening, data collection and intervention delivery were coordinated through a single centre (Amsterdam University Medical Centres).

### Ethics statement

Following approval by the Medical Ethics Committee of VU University medical centre (2019.605), the trial was registered in the Netherlands Trial Register: NL8157. Participants provided informed consent to participate and this was verbally reaffirmed during every interaction. All participants had capacity to consent to participate.

### Participants and setting

Recruitment of participant dyads took place via memory clinics, meeting centres, Alzheimer cafes, and healthcare organisations in the Netherlands between January 2020 and July 2022. In some cases the person with MCI/dementia was approached by the research or care professional, in other cases the informal caregiver was approached first, and both members of the dyad were informed and screened together. Participants (people with dementia/MCI or informal caregivers) could also self-refer via the project website. Eligible participants were community-dwelling people with an established diagnosis of MCI or mild dementia (Brief Cognitive Rating Scale [BCRS] 17–32), with an internet connection and an informal caregiver who also consented to participate. Exclusion criteria were: diagnosis of primary progressive aphasia, severe visual impairment, insufficient proficiency in Dutch language to provide informed consent, or simultaneous participation in another interventional study.

### Randomisation and masking

Participant dyads were enrolled by Rose-Marie Dröes (RMD) and David Neal (DN) and assigned to the FindMyApps or control arm by block randomisation using software in Castor EDC, with a target 1:1 allocation. Based on results of a pilot RCT,^10^ randomisation was stratified by: diagnosis (MCI or dementia), prior experience using a tablet (participant self-report; ‘Yes’ or ‘No’), and dyad cohabitation status (participant self-report; ‘Yes’ or ‘No’). Participants were not explicitly informed of trial arm assignment and the digital care-as-usual control condition was designed to weaken participants' recognition of and beliefs about trial arm assignment. However, neither participants nor investigators were formally blinded. Data analysis was conducted blind to trial arm.

### Interventions

The FindMyApps intervention was developed by a user-participatory design approach, by Saxion University of Applied Sciences, Amsterdam UMC and Radboud UMC, with support from software company Eumedianet.^8^ The intervention has three components: a tablet (running iPadOS or Android), the FindMyApps app (personalised app-selection tool, linked to a database of apps assessed as generally user-friendly in dementia, based on criteria developed through earlier research together with people with dementia and their caregivers),^13^ and training in the use of the tablet, and (for caregivers) in how to support people with dementia to learn to use the tablet. The app database comprises three broad categories of apps: “Around the house” (apps to support functional activities of daily living e.g. medication reminders, diaries), “Social contact” (apps for online communication e.g. video calling, instant messaging), and “Free time” (apps for engaging in hobbies and meaningful activities e.g. games, music, reminiscence activities). The database was updated periodically during the study, to replace defunct apps with appropriate alternatives. Training was given by one 60-min or two 30-min video calls (participant preference) by DN or masters students in clinical medicine, clinical neuropsychology and brain and cognitive sciences who had relevant training and experience with the interventions. The training was based on errorless learning, which facilitates instrumental learning through behaviour modelling, supervised practice and immediate error-correction.^14^ Errorless learning methods have been successfully applied in rehabilitation and occupational therapy for people with dementia, resulting in well-maintained learning of new procedural tasks. Caregivers were trained in implementation of errorless learning and were advised to practice with their counterpart during at least two 30-min sessions per week for four weeks. Participants were also provided with two training films (concerning use of the tablet and the FindMyApps app), and a printed guide covering the same topics. Participants could contact a ‘helpdesk’ with questions or problems, by telephone or email. The logic model relating the intervention to a theory of change and social health outcomes is presented in Supplementary Figure S1.

Control arm participants received digital care-as-usual, comprising a tablet of the same models provided in the experimental arm but without FindMyApps installed. Participants received one 60-min or two 30-min training sessions, and a printed handbook with information about the tablet. Instead of information about the FindMyApps app, the handbook contained a list of websites (such as the website of the Dutch dementia charity Alzheimer Nederland) where they could find apps recommended for people with dementia. This comprised types of apps similar to those in the FindMyApps database, related to support with activities of daily living, social contact and meaningful activities. Access to an online training film about the functions of the tablet, and to the helpdesk were also provided. The control arm training was not based on errorless learning.

### Outcomes and data collection procedure

Participant background characteristics were collected at baseline. Primary and secondary outcome data were collected from people with dementia/MCI via telephone interviews, with a researcher inputting data directly into a Castor EDC electronic database. Caregivers completed online questionnaires. Data were also collected regarding frequency of tablet use, and this was used to measure adherence (defined as engaging in at least two sessions a week with the tablet and/or FindMyApps for the first four weeks of the study).

Primary outcomes for the person with dementia/MCI were: self-management, measured by the Adult Social Care Outcomes Toolkit (ASCOT, range −0.17 to 1.00),^15^ and social participation, measured by frequency and diversity subscales on the Maastricht Social Participation Profile (MSPP, ranges 0–3 and 0–26 respectively).^16^ The primary caregiver outcome was sense of competence, measured by the Short Sense of Competence Questionnaire (SSCQ, range 1–7).^17^

Secondary outcomes for the person with dementia/MCI were: experienced autonomy, measured by the Experienced Autonomy instrument (EA, range 12–60, lower score indicates more autonomy),^18^ engagement in pleasurable activities, measured by a modified version of the Pleasurable Activities List (PAL, scale range 19–95),^19^ quality of life, measured on the five subscales of the Dementia Quality of Life Instrument (DQoL, each subscale range 1–5),^20^ and neuropsychiatric symptoms, measured by total symptom score on the Neuropsychiatric Inventory Questionnaire (NPI-Q, range 0–12).^21^ Secondary caregiver outcomes were: attitudes towards dementia, measured by the Approaches to Dementia Questionnaire (ADQ, range 19–95),^22^ and experience of providing care, measured by the Positive Experience Scale (PES, range 0–6).^23^

### Sample size

We aimed to include 150 dyads. Assuming a conservative estimate of within-subject correlation (0.3–0.5), approximately 15% fewer participants would be needed than that calculated using G∗Power version 3.1, for main effects MANOVA, for two dependent variables, two groups, alpha = 0.05 and power = 0.8, and a moderate effect size (eta-squared = 0.06).^24^ The required sample would therefore be around 134 dyads, allowing for 10% loss to follow-up.

### Statistical analysis

Data were cleaned and analysed with SPSS v.28. A senior researcher first removed trial arm identifiers. If more than 10% of participants were missing outcome data and data were missing completely at random (evaluated with Little's test), we planned to impute item-level data using expectation maximisation for participants missing less than 75% of items per instrument, and otherwise we planned to perform a completers-only analysis. Total or average scores were calculated for the pre and post-test outcome measures, following published guidance per instrument. Outcomes data were analysed based on the arm to which participants were randomised (intention-to-treat). Assumptions of statistical tests were checked prior to proceeding. ANCOVA is robust to violations of normality.

Participant background characteristics (demographics, diagnosis, BCRS score and presence of apathy) were described based on level of measurement (mean, standard deviation, range, frequencies and percentages). Differences between arms were tested by Student's t-test, Mann–Whitney U tests, or Chi–Square tests depending on level of measurement and distribution. Characteristics that differed significantly at alpha = 0.05 and were correlated with outcome measures at baseline were included in analyses as potential confounders.

### Effectiveness of FindMyApps compared to digital care-as-usual

Primary outcomes at three months were analysed by ANCOVA (self-management and caregivers sense of competence) or MANCOVA (social participation frequency and diversity), with trial arm as the independent variable, and baseline measurements as covariates, with a significance level of alpha = 0.05. Results of ANCOVAs were reported with estimated effect sizes (eta-squared, values of 0.01, 0.06, and 0.14 described as small, medium or large effects, respectively).^25^ We planned to analyse all secondary outcomes in a single MANCOVA, or if assumptions of MANCOVA were not met, by individual ANCOVAs - though the study was not adequately powered for multiple analyses by ANCOVA and no correction was therefore made for multiple testing.

### Effect modification and subgroup analyses

Five potential effect modifiers were defined prior to analysis and investigated by multiple linear regression, including group–modifier interaction terms for each outcome, except for neuropsychiatric symptoms measured by the NPI which produces a positively skewed discrete count, and was analysed by negative binomial regression. In addition to the three variables used to stratify randomisation (diagnosis, prior tablet use and cohabitation status), presence of apathy at baseline (yes or no) was investigated because, in the process evaluation accompanying this RCT, caregivers identified this as an implementation barrier.^11^ Age of the person with dementia/MCI was also investigated, as increasing age has been associated with less tablet use in dementia.^26^ Additionally, effect modification by whether or not participants adhered to the advised frequency of tablet use was also investigated. Subgroup analyses (two-way ANCOVA) were reported where the coefficient of the strongest effect modifier was statistically significant at alpha = 0.05. Non-significant results (0.05 < p < 0.10) were also described.

### Predictors of post-test outcomes

For primary outcomes, prediction linear regression models were constructed for post-test outcomes. A forward selection procedure was undertaken, starting with models including group and baseline score, and separately investigating the significance of background characteristics and adherence to advised training, iterating the model by sequentially adding the most significant predictor, up to a limit of p = 0.10. If trial arm was not a significant predictor, it was forced into the final model.

Further post-hoc analyses were undertaken based on observations made during the main analyses, namely paired samples t-tests with respect to social participation outcomes.

### Role of the funding source

No external funders had any role in study design, nor in the collection, analysis, or interpretation of data, writing the report or the decision to submit the paper for publication. All authors were involved in reviewing and editing the manuscript, had access to the study data and shared responsibility for the decision to submit for publication.

## Results

### Study population and baseline characteristics

**Fig. 1:**
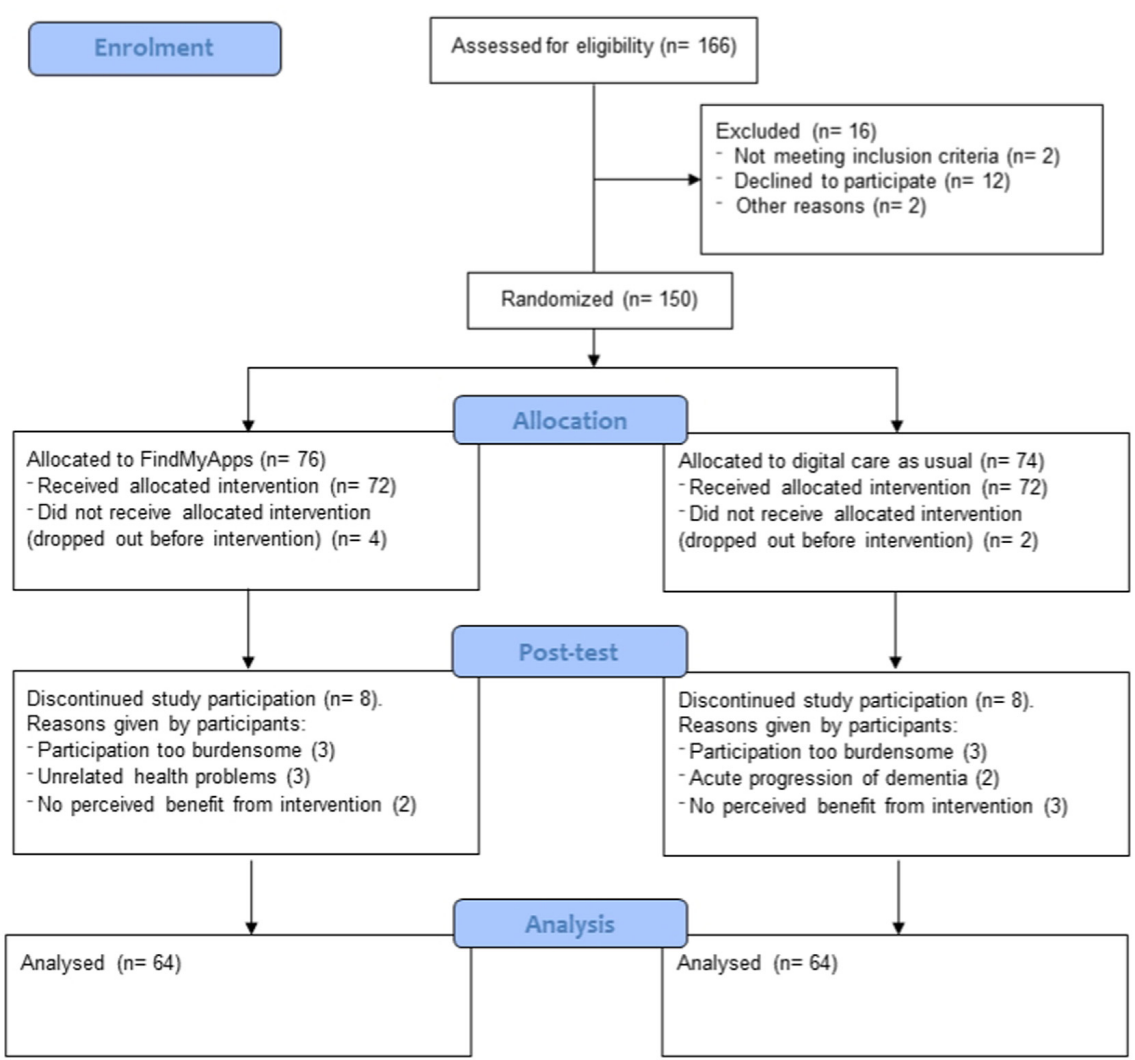
Participant flow from enrolment to analysis in the FindMyApps randomised controlled trial, demonstrating the number of participants assessed, randomized, allocated to each arm, analysed, and discontinuing study participation, with reasons given by participants for withdrawing.

Of 150 dyads randomised, 22 were lost to follow-up. Fewer than 10% of completers were missing item-level data, and completely at random (Little's test p = 1.000). All completers received the intervention to which they were assigned. Fig. 1 shows participant flows. Table 1 presents participants' background characteristics. Education level of people with dementia/MCI was included as a confounder in analysis of post-test PAL scores (baseline between-group difference, n = 122, χ^2^ 7.60, p = 0.022; correlation with baseline PAL rho = 0.24, p = 0.008).

**Table 1:** Background characteristics at baseline of experimental and control arm participants.

	Experimental (n = 64)	Control (n = 64)	Difference test	p
**Persons with dementia**				
Mean age (SD) [range]	72.61 (9.51) [50–95]	72.06 (9.22) [53–93]	t = 0.33	0.742
<65, n (%)	13 (20)	16 (25)		
65–74, n (%)	21 (33)	20 (31)		
75–84, n (%)	25 (39)	21 (33)		
85+, n (%)	5 (8)	7 (11)		
Sex, n (%)			χ^2^ = 0.13	0.722
Male	35 (55)	37 (58)		
Female	29 (45)	27 (42)		
Diagnosis, n (%)			χ^2^ = 0.53	0.465
MCI	22 (34)	26 (41)		
Mild Dementia	42 (66)	38 (59)		
Alzheimer	28 (67)	18 (47)		
Vascular	8 (19)	1 (3)		
Lewy body	1 (2)	9 (24)		
Frontotemporal	1 (2)	2 (5)		
Other	4 (10)	8 (21)		
Mean BCRS (SD) [range]	23.72 (4.42) [17–32]	23.48 (4.33) [13–33]	t = 0.30	0.762
Apathy present at baseline, n (%)	34 (53)	33 (52)	χ^2^ = 0.03	0.860
Used tablet before study, n (%)	40 (63)	40 (63)	χ^2^ = 0.00	1.000
Highest education, n			χ^2^ = 7.60	0.022
Primary	12 (19)	16 (25)		
Secondary	11 (17)	22 (34)		
Tertiary	41 (64)	26 (41)		
**Caregivers**				
Mean age (SD) [range]	64.48 (11.65) [25–87]	61.31 (14.58) [17–88]	U = 1817.00	0.271
<65, n	30 (47)	40 (63)		
65–74, n	22 (34)	11 (17)		
75–84, n	12 (19)	12 (19)		
85+, n	0 (0)	1 (2)		
Sex, n (%)			χ^2^ = 0.16	0.689
Male	18 (29)	16 (24)		
Female	46 (71)	48 (76)		
Highest education, n (%)			χ^2^ = 0.39	0.824
Primary	8 (13)	10 (16)		
Secondary	23 (36)	24 (38)		
Tertiary	33 (52)	30 (47)		
Relationship with PwD, n (%)			χ^2^ = 0.05	0.997
Partner	47 (73)	46 (72)		
Son (in-law)/daughter (in-law)	11 (17)	12 (19)		
Sibling	1 (2)	1 (2)		
Other	5 (8)	5 (8)		
Cohabit with PwD, n (%)	48 (75)	48 (75)	χ^2^ = 0.00	1.000

Note: SD = standard deviation; PwD = person with dementia; MCI = Mild cognitive impairment; BCRS = Brief Cognitive Rating Scale.

### Use of interventions during the study

Detailed observations regarding use of the tablet and FindMyApps app have been reported elsewhere.^11^ Adherence rates to the advised training scheme were 66.7% in the experimental arm and 64.5% in the control arm (n_tot_ = 122, chi-squared = 0.06, p = 0.80). No harms associated with the interventions were identified.

### Effectiveness of FindMyApps compared to digital care-as-usual

Table 2 presents results of the primary outcomes ANCOVAs and MANCOVA. A significant, medium effect of the FindMyApps intervention on caregivers’ post-test SSCQ score, controlled for baseline SSCQ score, was found (F [1,123] = 7.01, p = 0.009, η^2^ = 0.054). There were no other significant effects of FindMyApps on post-test outcomes. Sensitivity analyses conducted on the dataset with missing item-level data imputed by expectation maximisation where less than 75% of items per instrument were missing did not result in changes in the direction or significance of between-group differences on the ASCOT (F [1,123] = 0.29, p = 0.589, η^2^ = 0.002) or MSPP (F [1,112] = 0.27, p = 0.768, η^2^ = 0.005). There was no missing item-level data on the SSCQ.

**Table 2:** Results of ANCOVA and MANCOVA analyses of primary and secondary outcomes.

Outcome [Instrument] (scale range)^a^	n	Mean experimental T0	Mean control T0	Mean experimental T3	Mean control T3	Adjusted post-test means experimental/control	F	p	η^2^
**Primary outcomes**									
*Person with dementia/MCI*									
Self-management [ASCOT] (−0.17 to 1.00)	120	0.82	0.82	0.83	0.82	0.83/0.82	0.12	0.727	0.001
Social participation [MSPP frequency] (0–3)	114	2.46	2.35	2.63	2.49	2.61/2.51	0.24	0.784	0.005
Social participation [MSPP diversity] (0–26)		12.10	12.34	13.38	12.73	13.10/12.57			
*Caregiver*									
Sense of competence [SSCQ] (0–7)	126	4.95	5.05	5.06	4.37	5.08/4.34	7.01	0.009	0.054
**Secondary outcomes**									
*Person with dementia/MCI*									
Experienced autonomy [EA] (12–60)	124	31.54	31.81	30.31	31.95	30.39/31.87	2.36	0.127	0.019
Engagement in pleasurable activities [PAL] (19–95)^b^	111	45.58	44.92	45.82	45.42	45.43/45.59	0.02	0.903	0.000
Quality of life [DQoL belonging] (1–5)	125	3.84	3.60	3.83	3.68	3.77/3.75	0.05	0.824	0.000
Quality of life [DQoL positive affect] (1–5)	124	3.56	3.51	3.61	3.56	3.60/3.57	0.10	0.758	0.001
Quality of life [DQoL negative affect](1–5)	125	3.63	3.57	3.70	3.52	3.69/3.53	3.77	0.054	0.030
Quality of life [DQoL negative affect](1–5)	125	2.43	2.49	2.38	2.43	2.40/2.41	0.00	0.972	0.000
*Caregiver*									
Attitudes to dementia [ADQ] (19–95)	126	65.28	66.84	65.65	65.51	66.18/64.98	2.46	0.119	0.020
Caregiver experience of care [PES] (0–6)	126	3.11	3.16	3.22	3.21	3.25/3.18	0.07	0.791	0.001

Note: MSPP = Maastricht Social Participation Profile, ASCOT = Adult Social Care Outcomes Toolkit, SSCQ = Short Sense of Competence Questionnaire, EA = Experienced Autonomy questionnaire, PAL = Pleasurable Activities List, DQoL = Dementia Quality of Life Instrument, ADQ = Attitudes to Dementia Questionnaire, PES = Positive Experiences Scale.

a In each case most desirable score is underlined.

b Main effect is reported, controlled for confounding by education level of the person with dementia/MCI. The MSPP subscales were analysed with MANCOVA.

Assumptions of linear correlation between outcomes and covariates for secondary outcomes MANCOVA were not met. No differences on individual ANCOVAs were statistically significant at alpha = 0.05. There was an overall pattern of small effect sizes in favour of FindMyApps, most notably on the DQoL positive affect subscale. Owing to heterogeneity of regression slopes on the DQoL sense of aesthetics subscale, a modified Johnson-Neyman procedure was undertaken instead of ANCOVA, to determine the relationship between pre-test and post-test scores.^27^ Participants who received the FindMyApps intervention had higher post-test sense of aesthetic scores than the control group if their baseline score was below 3.50, and lower post-test sense of aesthetics scores than the control group if their baseline score was above 3.50 (scale range 1–5). The differences between trial arms were statistically significant in favour of the FindMyApps intervention at pre-test scores below 2.52 (n = 9), and in favour of the control arm at scores above 4.46 (n = 22).

### Effect modification and subgroup analyses

Assumptions of multiple regression approaches were met. Age of the person with dementia/MCI was not linearly related to post-test outcomes and was analysed as a dichotomous variable around the median (73 years).

For primary outcomes, no significant interactions were found, meaning that outcomes did not differ significantly between the defined subgroups. When stratified for pre-defined modifiers, non-significant results (0.05 < p < 0.10) were identified, regarding self-management measured by the ASCOT (group by prior tablet use) in favour of FindMyApps users with prior tablet experience (p = 0.094), and social participation on the MSPP frequency scale (group by diagnosis) in favour of FindMyApps users diagnosed with MCI compared to dementia (p = 0.093), suggesting that tablet experience and diagnosis might modify the effect of FindMyApps on self-management and social participation, respectively.

On three secondary outcomes significant differences between subgroups were found (Table 3): People with MCI experienced more engagement in pleasurable activities with FindMyApps, and people with apathy at baseline had better outcomes on the DQoL Sense of Belonging subscale and Positive Affect subscales with FindMyApps. Further non-significant results were identified in the secondary outcomes experienced autonomy, measured on EA (group by age) in favour of older users of FindMyApps (p = 0.057), neuropsychiatric symptoms, measured on NPI (group by diagnosis) in favour of FindMyApps users who had the diagnosis MCI rather than dementia (p = 0.071), and engagement in pleasurable activities, measured with the PAL (group by prior tablet use) in favour of FindMyApps users with prior tablet experience (p = 0.086), suggesting that age, diagnosis and tablet experience potentially modify the effect of FindMyApps on autonomy, neuropsychiatric symptoms and engagement in pleasurable activities respectively.

**Table 3:** Results of two-way ANCOVAs on secondary outcome measures.

Outcome [instrument] (scale range^a^)	n	Mean exp. T0	Mean cont. T0	Mean exp. T3	Mean cont. T3	Adjusted post-test means exp/cont^a^	F	p	η^2^
**Person with dementia or mild cognitive impairment**									
Engagement in pleasurable activities [PAL] (19–95)	111						0.53	0.470	0.005
MCI		50.18	48.29	50.84	46.52	48.05/44.24			
Dementia		43.05	42.67	43.24	44.74	44.45/46.39			
Quality of life [DQoL belonging] (1–5)	125						0.05	0.851	0.000
No apathy at T0		3.83	3.71	3.64	3.87	3.59/3.88			
Apathy at T0		3.84	3.49	4.00	3.50	3.94/3.61			
Quality of life [DQoL positive affect] (1–5)	125						3.77	0.058	0.030
No apathy at T0		3.71	3.63	3.66	3.66	3.59/3.65			
Apathy at T0		3.57	3.52	3.74	3.38	3.77/3.43			

Where effect modification was found, the result for main effect of trial arm is reported, together with estimated marginal means.

Note: PAL = Pleasurable Activities List, DQoL = Dementia Quality of Life Instrument.

a In each case most favourable score is underlined.

For the NPI, the rate ratio for post-test mean total symptoms between experimental and control arms, adjusted for pre-test mean total symptoms, was 0.50 for people with MCI (n = 47, 95% CI 0.25–0.97), and for people with dementia 1.05 (n = 79, 95% CI 0.65–1.71). This indicates that people with MCI who received FindMyApps appeared to have fewer neuropsychiatric symptoms at post-test than those in the control arm, whereas there was no significant difference for people with dementia.

Adherence to the advised training schema was not a significant effect modifier with respect to any of the measured outcomes.

### Predictors of post-test outcomes

Prediction multiple linear regression models for post-test outcomes and their goodness of fit are shown in Table 4. Age and diagnosis may be relevant predictors of post-test outcomes for the person with dementia/MCI, regardless of intervention. Apathy at baseline and education level of the person with dementia/MCI may be additional predictors of caregiver outcomes.

**Table 4:** Prediction linear regression models with respect to primary outcomes.

	Regression coefficients		p	95% Confidence Interval	
	B	Std. Error		Lower Bound	Upper Bound
**Model forself-management(ASCOT total score T3) r2 = 0.309**					
(Constant)	0.328	0.071	<0.001	0.187	0.469
Experimental arm	0.008	0.022	0.727	−0.036	0.051
ASCOT total score at T0	0.610	0.085	<0.001	0.443	0.778
**Model for social participation (MSPP frequency score T3) r2 = 0.409**					
(Constant)	1.256	0.255	<0.001	0.750	1.762
Experimental arm	0.114	0.176	0.519	−0.235	0.463
MSPP frequency score at T0	0.630	0.077	<0.001	0.478	0.782
Person with dementia age 73 years or older	−0.309	0.178	0.086	−0.661	0.044
**Model for social participation (MSPP diversity score T3) r2 = 0.388**					
(Constant)	7.437	1.249	<0.001	4.961	9.912
Experimental arm	0.752	0.749	0.318	−0.733	2.236
MSPP diversity score at T0	0.622	0.084	<0.001	0.455	0.788
Person with dementia age 73 years or older	−2.013	0.756	0.009	−3.512	−0.513
Diagnosis dementia vs MCI	−1.330	0.797	0.098	−2.910	0.251
**Model for caregiver sense of competence (SSCQ total score T3) r2 = 0.466**					
(Constant)	2.328	0.471	<0.001	1.395	3.260
Experimental arm	0.575	0.270	0.035	0.040	1.110
SSCQ total score at T0	0.569	0.075	<0.001	0.420	0.718
Person with dementia completed tertiary education	0.716	0.276	0.011	0.170	1.262
Apathy reported at T0	−1.022	0.274	<0.001	−1.565	−0.480

In all cases higher scores are more favourable, so variables with positive coefficients predict better outcomes and those with negative coefficients predict worse outcomes.

Note: ASCOT = Adult Social Care Outcomes Toolkit; MSPP = Maastricht Social Participation Profile; SSCQ = Short Sense of Competence Questionnaire.

### Post-hoc analyses

Post-test MSPP frequency and diversity scores did not differ between trial arms at post-test, but small increases from baseline were observed in both arms (see Table 5).^25^ The increase in diversity of social participation of experimental arm participants was statistically significant.

**Table 5:** Paired samples t-tests comparing post-test with baseline scores for frequency and diversity of social participation, per trial arm.

	n	Pre-test mean	Post-test mean	t	p	Cohen's d
MSPP frequency (range 0–3)^a^						
Experimental arm	57	2.46	2.63	1.15	0.253	0.153
Control arm	57	2.31	2.43	1.00	0.322	0.132
MSPP diversity (range 0–26)^a^						
Experimental arm	57	12.12	13.42	2.05	0.046	0.271
Control arm	57	12.29	12.63	0.81	0.424	0.107

Note: MSPP = Maastricht Social Participation Profile.

a In each case most desirable score is underlined.

## Discussion

The primary goal of this RCT was to evaluate the effectiveness of FindMyApps for supporting self-management and social participation of people with mild dementia or MCI and sense of competence of their informal caregivers, compared to digital care-as-usual. Overall, there were no statistically significant differences between intervention arms on primary or secondary outcomes for people with dementia/MCI. Caregivers who received FindMyApps had moderately higher sense of competence at three months than those receiving digital care-as-usual. Investigation of effect modifiers suggested a more beneficial effect of FindMyApps on neuropsychiatric symptoms and engagement in pleasurable activities for people with a diagnosis of MCI, and for people with mild dementia/MCI with apathy at baseline on quality of life outcomes. Younger age of the person with mild dementia/MCI, a diagnosis of MCI, the person with mild dementia/MCI having completed tertiary education, and not experiencing apathy at baseline seemed to predict better post-test outcomes, regardless of the intervention received. Small improvements in social participation between baseline and post-test were seen in both arms, with the increase in diversity of social activities with FindMyApps being statistically significant.

The absence of statistically significant group-level effects of FindMyApps on outcomes for people with mild dementia/MCI, and on the other hand, the identification of effect modifiers (diagnosis and experiencing apathy influenced intervention outcomes), demonstrate that the intervention was not straightforwardly effective for all people with mild dementia/MCI, suggesting the need for more tailored interventions. The intervention and how it was implemented in this study were identical for all participants, whereas certain subgroups of participants may have benefited from a more personalized intervention or implementation process. This was also reflected in results of a parallel mixed-methods process evaluation.^11^

Another reason that no effects on a group level were found may be because FindMyApps appeared primarily associated with higher quality tablet interactions but not quantitatively more tablet use or adoption.^11^ Many factors can limit technology adoption by people with dementia, including lack of perceived need for a device, or unwillingness or inability to invest time in learning technology.^5^^,^^7^^,^^11^ Adherence rates in this study were similar in both arms (about 65%) and higher than the 56% average reported for eHealth interventions for non-communicable disease management.^28^ However, the average frequency of use, or the three-month duration of the follow-up period, may have been insufficient to observe significant improvements in outcomes. Finally, the impact of individual apps from the FindMyApps database on self-management, social participation and other outcomes has not been investigated and it may be that the apps used by participants during the study did not greatly impact the measured outcomes.

Real group-level differences may not have been detected due to measurement of post-test outcomes at a single point in time, potentially masking trends or changes in variability in outcomes over time. We also note a ceiling effect measuring self-management with the ASCOT. This has been previously reported,^29^ and may be more notable in this study as participants at baseline had the motivation and means to participate, and people with MCI (34% in the FindMyApps group and 41% in the control group) had relatively limited functional impairments. Since previous research demonstrated a dearth of suitable instruments for measuring self-management in this context,^30^ a new instrument may be required for future studies.

The positive effect of FindMyApps on Caregivers’ sense of competence may be because the intervention was easier for them to teach and easier to learn for the person with mild dementia/MCI than a standard tablet, and was rated by caregivers as more fun to use.^11^ Even if the intervention did not impact measured outcomes for all people with mild dementia/MCI, caregivers in general may have felt more competent in their role as facilitators if the learning process was easier. In a pilot trial, no effect of FindMyApps on sense of competence was found, perhaps because at that time caregivers were more negative about the quality of training they received and may therefore have experienced more burden in implementing the intervention.^10^ For this study the training was improved and was positively evaluated by caregivers.^11^

Previously it was unclear which participants would benefit more from FindMyApps: those with prior tablet experience, high caregiver support, and MCI rather than dementia (features facilitating engagement with a tablet) or those experiencing the greatest barriers to benefitting from digital care-as-usual. People with MCI seemed to be more engaged in pleasurable activities with the FindMyApps intervention and had fewer neuropsychiatric symptoms compared to baseline, suggesting that some additional cognitive work was required to benefit from FindMyApps, and those with more cognitive capacity therefore benefitted more. Indeed, a diagnosis of MCI predicted better post-test outcomes for both members of the dyad, regardless of intervention. A review of studies with other digital interventions similarly found greater benefits for people with MCI than dementia [6]. Apathy was described by caregivers as a barrier to implementing interventions, and predicted worse post-test social participation overall, but was associated with better quality of life outcomes of the person with mild dementia/MCI (sense of belonging and positive affect) with FindMyApps. Participants described FindMyApps as a more focussed experience than digital care-as-usual because specific apps were recommended.^11^ This may be more stimulating for those with apathy but of less added value for those not experiencing apathy.

This study was not designed to investigate longitudinal changes in social participation. However, observed small increases in social participation are in line with studies of other digital interventions, which also found weak evidence of positive effects on social participation.^5^^,^^6^

This study overcame three methodological limitations of previous RCTs evaluating digital technology for social health in dementia.^6^ The study was adequately powered to detect medium effects of the intervention on primary outcomes, the control condition was an active digital care-as-usual intervention, and the statistical analysis was described in sufficient depth to allow replication. A process evaluation also facilitated interpretation of trial results.^9^^,^^11^

On the other hand, the study was not sufficiently powered to draw strong conclusions about effect modifiers or subgroup analyses. Imputation of missing data by expectation maximisation has the theoretical potential to bias results of analyses but this is unlikely to have had an important impact on our results because less than 10% of data were imputed and data were missing completely at random (MCAR). It was still not possible to fully blind participants and investigators to trial arm assignment during the study. The Pleasurable Activities List self-report instrument has not previously been validated specifically in dementia, and it is possible that impaired recall may have affected these results. The methods and intervention were both impacted by the COVID-19 pandemic, with data collection and the tablet training component of the interventions taking place online, rather than face-to-face, as originally planned. This may have negatively influenced outcomes and limits generalisation of the results, since in future we would anticipate participants being offered face-to-face training in addition to, or instead of, online training. Another limitation to generalising these results is the likelihood of sampling bias created by participants self-referring or volunteering in the study, although most of the participants were invited to participate via memory clinics and care and welfare organisations.

Whilst certain participant characteristics, such as diagnosis and experiencing apathy, were associated with more benefit from FindMyApps than digital care-as-usual, no evidence was found that FindMyApps is in general more directly beneficial than a standard tablet for people with mild dementia/MCI. This study demonstrates that despite the prevalence of technological interventions specifically designed for people with dementia, at a group level generic alternatives could be as effective. However, this may also be seen as evidence of the limitations of RCT designs, which ideally require large, homogenous samples of participants who experience a single standardised intervention, whereas in practice sample sizes tend to be small and/or heterogeneous, and individual needs, preferences and abilities may differ, requiring tailored implementation and evaluation of eHealth interventions.

Regarding tablet-based interventions in routine care, FindMyApps was shown to be better for caregivers’ sense of competence than a normal tablet. This means that FindMyApps has greater potential to support caregivers to provide better care, for longer, bringing indirect benefits for the person with dementia/MCI in the form of delayed nursing home admissions.^1^^,^^17^ FindMyApps may therefore be preferable to a normal tablet, particularly for people with MCI and people experiencing apathy.

Future research should be conducted with a more representative sample of the general population, with an alternative instrument to measure self-management, and with a longer follow-up period. Evaluating variations on the individual components of the intervention (tablet, functional components of the FindMyApps app, and training), and investigating the effect of individual apps in the FindMyApps database, would also help to further understand the mechanisms of impact of the intervention and what may be most valuable for users in practice. The effectiveness of face-to-face compared to online tablet training should be further evaluated. The cost-effectiveness of the FindMyApps intervention is currently being evaluated. People with mild dementia/MCI and their caregivers should consider the potential of FindMyApps or other tablet interventions for supporting social participation, particularly for younger people, and those with a diagnosis of MCI. Older users and those with a diagnosis of mild dementia may benefit from additional support to learn to use the tablet, and may require a form of intervention more tailored to their needs. Future research with FindMyApps should evaluate such alternatives.

No statistically significant differences were found on a group level in outcomes following the FindMyApps intervention compared to digital care as usual for community-dwelling people with mild dementia/MCI. On a subgroup level differences in favour of the FindMyApps group were found for pleasurable activities and neuropsychiatric symptoms for people with MCI, and sense of belonging, and positive affect for people experiencing apathy. The greatest benefit from the FindMyApps intervention is better support for the informal caregiver. Pending results of an economic evaluation, these results may support implementation of FindMyApps in routine care, for the purposes of supporting social participation of people with dementia/MCI, and sense of competence of informal caregivers. Implementation of tablets in general may be more beneficial for people with a diagnosis of MCI and younger people with mild dementia/MCI. For people with a diagnosis of mild dementia and older people with mild dementia/MCI, better tailored interventions, implementation and outcome measures are needed.

## Contributors

DN, TE, KD, MG and RD conceived of the study and developed the methodology. DN, TE, KD, MM and RD acquired funding and carried out the investigation. MZ also supported investigation. MZ and RD provided additional resources. DN and TE were further responsible for data curation and formal analysis. DN and RD were responsible for project administration. TE, KD, EF, MG, MM and RD provided supervision. DN prepared the original draft of the manuscript.

## Data sharing statement

All of the individual participant data collected during the trial, after de-identification, and the study protocol will be made available from immediately following publication to researchers who provide a methodologically sound proposal, to achieve aims in the approved proposal. Proposals should be directed to rm.droes@amsterdamumc.nl; to gain access, data requestors will need to sign a data access agreement.

## Declaration of interests

RMD is Chair of the MEETINGDEM Network, which aims to disseminate the concept of the Meeting Centers Support Programme for people with dementia, and facilitate knowledge exchange, exchange of experiences, and international collaboration in applied research, and since January 2023 of the non-profit JAIN (Joint Artificial Intelligence Network) Foundation, in both roles unremunerated.
