# Supplementary material for: FindMyApps compared with usual tablet use to promote social health of community-dwelling people with mild dementia and their informal caregivers: a randomised controlled trial
Source: eClinicalMedicine. 2023 Aug 30;63:102169. doi: 10.1016/j.eclinm.2023.102169 (PMC10480525; doi:10.1016/j.eclinm.2023.102169)

Supplementary material

Figure S1. Logic model relating the FindMyApps intervention to behavioural theory of change and social health outcomes. Note: *PwD = person with dementia; CG = caregiver.*

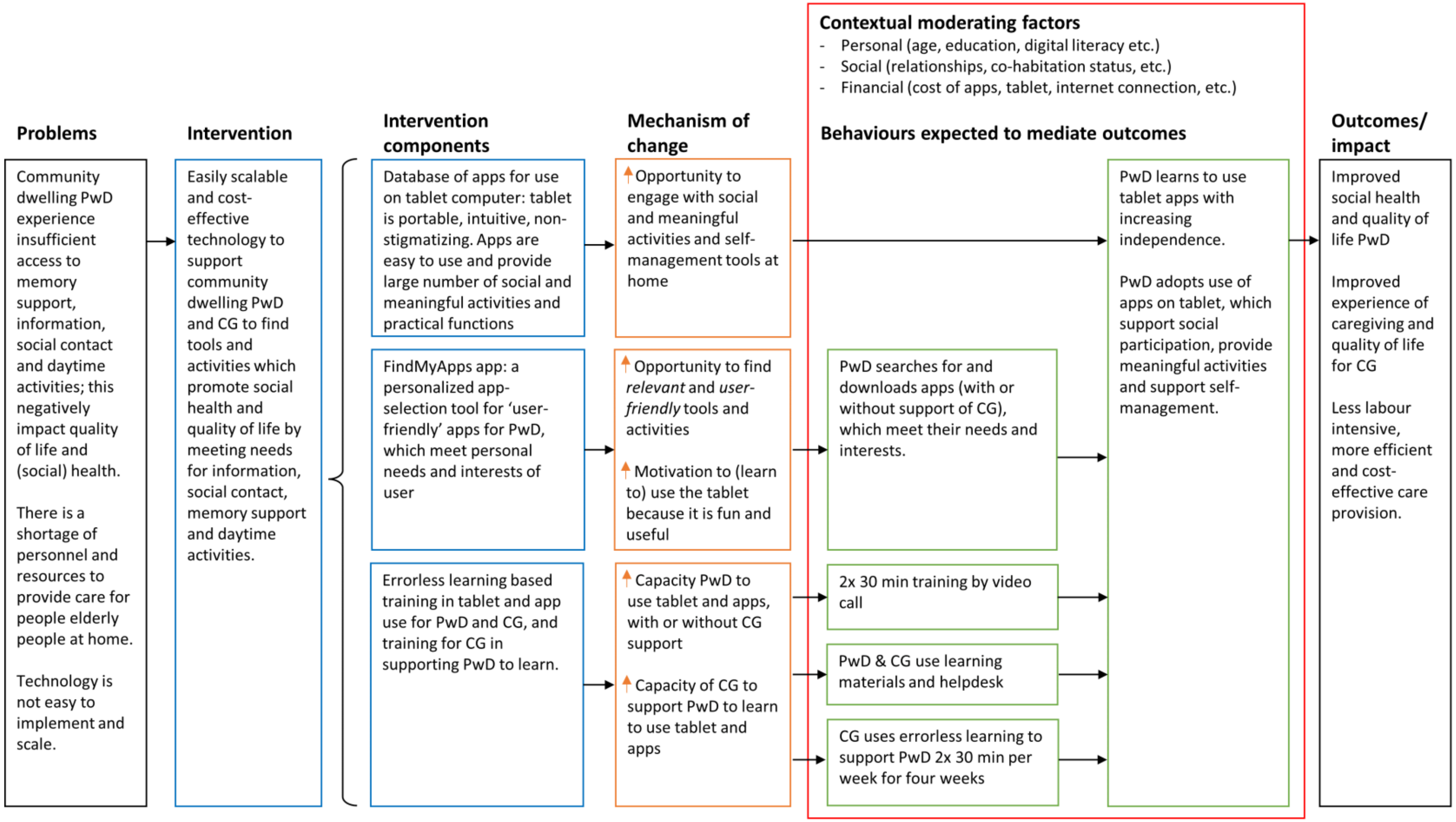

Supplement: Supplementary material [file mmc1.pdf]
